# A Biochemically “Silent” Aortocaval Paraganglioma in a 19‐Year‐Old Female Causing Intraoperative Hypertensive Crisis: A Rare Case Report

**DOI:** 10.1002/ccr3.72603

**Published:** 2026-04-24

**Authors:** Abdul Basit, Tooba Yaqoob, Eyman Farrukh, Abdullah Iftikhar, Muhammad Usman Ali Khan, Majeed Haq

**Affiliations:** ^1^ King Edward Medical University Lahore Pakistan; ^2^ Fatima Memorial Hospital Lahore Pakistan; ^3^ Shaheed Suhrawardy Medical College and Hospital Dhaka Bangladesh

**Keywords:** aortocaval mass, catecholamine, extra‐adrenal pheochromocytoma, hypertensive crisis, non‐functional tumor, paraganglioma

## Abstract

Paragangliomas may appear biochemically silent, yet still behave as functional tumors with severe intraoperative consequences. Normal preoperative catecholamine screening does not exclude the risk of hypertensive crisis during surgery. Thorough biochemical evaluation, high clinical suspicion, and perioperative preparedness with multidisciplinary planning are essential to prevent life‐threatening complications.

## 1 Background

Paragangliomas are rare neuroendocrine tumors arising from extra‐adrenal chromaffin cells. They often secrete catecholamines (functional), but a minority are clinically silent, which can complicate diagnosis. We report a 19‐year‐old female with 6 months of vague abdominal pain. Imaging revealed a 6 cm aortocaval mass suspected to be an extra‐adrenal paraganglioma. Initial biochemical screens for catecholamine excess were normal, suggesting a non‐functional tumor. She underwent exploratory laparotomy without preoperative adrenergic blockade. Intraoperatively, she experienced a hypertensive crisis during induction, and intravenous vasodilators were required. The mass was resected successfully. Histopathology confirmed paraganglioma with typical neuroendocrine features. Postoperatively, plasma‐free normetanephrine levels returned markedly elevated, retrospectively indicating a functional tumor. This case highlights the diagnostic challenges of “silent” paragangliomas and the importance of thorough biochemical evaluation and perioperative preparedness. Even tumors with normal initial catecholamine tests may trigger life‐threatening intraoperative hypertension, underscoring the need for caution and multidisciplinary management.

## 2 Introduction

Paragangliomas are rare neuroendocrine tumors derived from neural crest cells, with an annual incidence estimated around 2–8 per million population [1]. They constitute the extra‐adrenal counterpart of pheochromocytomas, accounting for roughly 10%–15% of all catecholamine‐secreting tumors [1, 2]. Sympathetic paragangliomas typically arise in the abdomen or pelvis and often produce excess norepinephrine, leading to the classic triad of episodic headache, palpitations, and sweating, as well as sustained or paroxysmal hypertension [2, 3]. However, a small subset (approximately 10%) of paragangliomas are clinically nonfunctional (silent), secreting little to no catecholamine; these usually present with only vague symptoms (e.g., abdominal pain) or are discovered incidentally on imaging [3]. The clinical presentation is therefore highly variable—ranging from asymptomatic masses to catastrophic hypertensive crises—which makes preoperative diagnosis challenging [2]. Furthermore, these tumors are exceedingly rare and often overlooked in the differential diagnosis of abdominal masses. Recognizing a paraganglioma before surgery is critical, as undiagnosed functional tumors pose significant perioperative risks: the stress of induction or tumor manipulation can precipitate life‐threatening surges in blood pressure if appropriate precautions are not in place [4]. Advances in preoperative management, particularly the use of alpha‐adrenergic blockade, have dramatically improved surgical outcomes for pheochromocytomas/paragangliomas, reducing intraoperative mortality from ~40% in the mid‐20th century to under 3% in recent decades [3, 5]. We report a case of an extra‐adrenal (aortocaval) paraganglioma in a young female patient, initially presumed to be nonfunctional based on biochemical testing. The case underlines the diagnostic complexity and perioperative challenges associated with these rare tumors, especially when biochemical tests are misleading.

## 3 Case History/Examination

A 19‐year‐old unmarried female, resident of Lahore, presented to the surgical outpatient department with complaints of intermittent abdominal pain for the past 6 months. The pain was diffuse in nature, not localized to any quadrant, and described as vague, dull, and gradually progressive. It was mild to moderate in intensity, non‐colicky, and episodic, with each episode lasting several hours before subsiding spontaneously. There was no associated fever, nausea, vomiting, bowel or urinary disturbances, or menstrual irregularities. She denied any history of headache, palpitations, diaphoresis, flushing, orthostatic dizziness, anxiety attacks, or episodic hypertension. Over the course of her symptoms, she reported an unintended weight loss of approximately 5 kg, which she attributed to poor oral intake during pain episodes. She sought symptomatic relief from private clinics, where she was intermittently prescribed analgesics. There was no history of prior hospitalizations, surgeries, or chronic illnesses, and she had no history of tobacco, alcohol, or drug use. Family history was noncontributory, with no known cases of hypertension, endocrine disorders, or genetic syndromes such as multiple endocrine neoplasia (MEN), von Hippel–Lindau disease (VHL), or neurofibromatosis type 1 (NF1).

On general physical examination, she appeared healthy, alert, and oriented. Her vital signs remained within normal limits throughout her hospital stay, with a blood pressure ranging from 100/70 to 120/80 mmHg, pulse rate of 74/min, and she was afebrile and clinically euvolemic. There were no signs of pallor, icterus, lymphadenopathy, or pedal edema. Cardiovascular, respiratory, and neurological examinations were unremarkable. Her abdomen was soft and non‐tender with no palpable mass or visceromegaly, and bowel sounds were normally audible.

## 4 Differential Diagnosis, Investigations, and Treatment

- *Abdominal mass*—Could be a benign or malignant tumor, including lymphoma or gastrointestinal stromal tumor (GIST).
- *Ovarian cyst or tumor*—Considering the patient's age and abdominal complaints
- *Gastrointestinal issues*—Such as peptic ulcer disease or irritable bowel syndrome.
- *Pheochromocytoma*—Though less likely given the absence of classic symptoms, it must still be considered due to the potential for episodic hypertension.
- *Renal pathology*—Cysts or neoplasms could also cause abdominal discomfort.

Initial laboratory investigations were largely within normal ranges. Hemoglobin was 13.1 g/dL, total leukocyte count was 8400/μL with a normal differential count, and platelet count was adequate. Liver function tests revealed mildly elevated transaminases (ALT 46 IU/L, AST 57 IU/L), with normal bilirubin and alkaline phosphatase. Renal profile and serum electrolytes were within normal limits. Tumor markers and thyroid function tests were not indicative of any active pathology.

**FIGURE 1:**
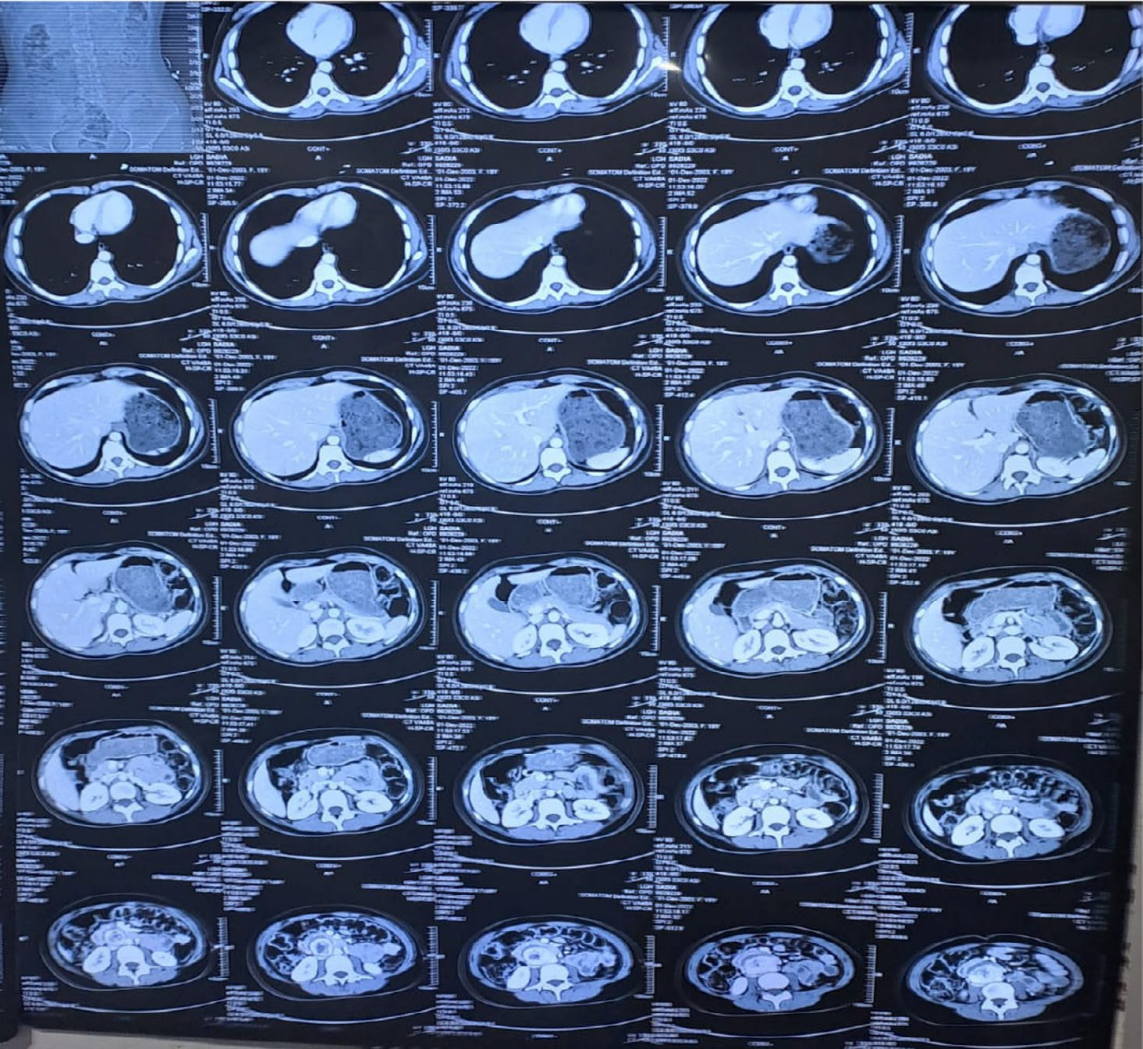
CT Abdomen with IV contrast showing the paraganglioma.

**FIGURE 2:**
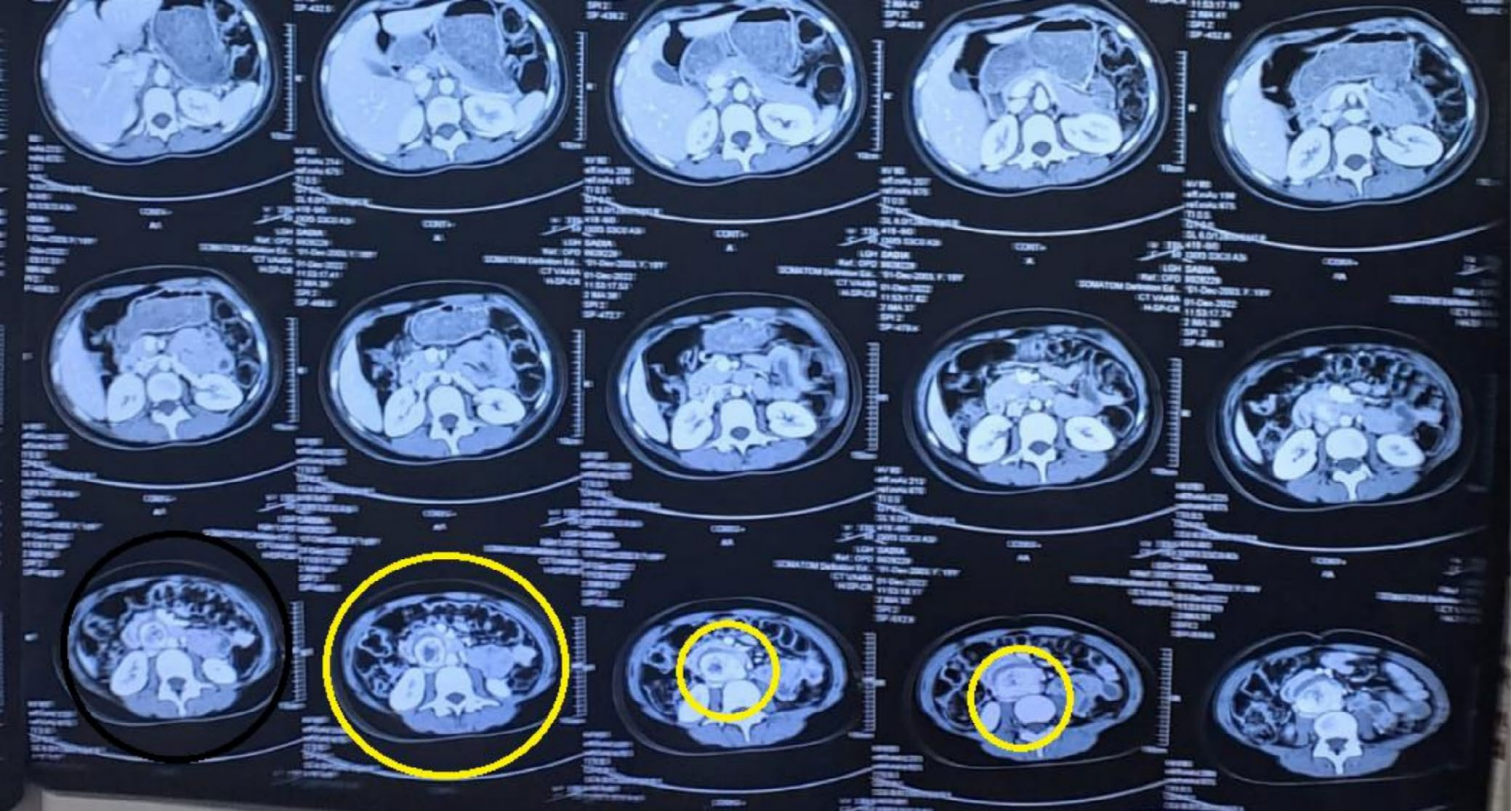
The encircled section in CT Abdomen with IV contrast showing the mass.

A baseline abdominal ultrasound revealed a well‐defined, solid‐looking lesion measuring approximately 4.8–4.9 cm in the mid‐abdomen, situated deep to the fascia. A repeat ultrasound performed 6 weeks later showed a hypoechoic, heterogeneously textured mass measuring 6.0 × 2.9 cm in the para‐aortic region above the umbilicus, with minimal internal vascularity on color Doppler. Contrast‐enhanced computed tomography (CT) of the abdomen demonstrated a 6.1 × 4.0 × 3.0 cm heterogeneously enhancing soft tissue mass in the aortocaval region, just below the inferior mesenteric artery and anterior to the L3–L4 vertebral bodies. The mass was partially encasing the aorta and compressing and displacing the inferior vena cava (IVC) and adjacent small bowel loops. A surrounding edematous rim and central necrotic area were noted as shown in Figures 1 and 2.

Magnetic resonance imaging (MRI) of the abdomen confirmed a lobulated retroperitoneal lesion measuring 6.0 × 3.2 × 3.7 cm between the aorta and IVC, hyperintense on T2 and STIR sequences and hypointense on T1, with heterogeneous post‐contrast enhancement and internal cystic and necrotic components. These radiological features were highly suggestive of a paraganglioma originating from the organ of Zuckerkandl region.

Despite the absence of classic catecholamine‐related symptoms, a 24‐h urinary metanephrine assay was conducted due to the radiologic suspicion. The result returned within normal limits at 120.4 mcg/24 h (reference: 22–345 mcg), suggesting a nonfunctional tumor. Given the mass's size and compressive effects, a USG‐guided percutaneous biopsy was performed, which revealed nests and ribbons of polygonal cells with eosinophilic cytoplasm and uniform round nuclei in a fibrovascular stroma, arranged in the characteristic “zellballen” pattern. Immunohistochemistry demonstrated strong diffuse positivity for chromogranin A and synaptophysin, with S‐100 positivity highlighting the sustentacular framework and GATA3 positivity, consistent with extra‐adrenal paraganglioma.

The patient was scheduled for elective surgical resection under general anesthesia. Based on the presumed nonfunctional status of the tumor, no preoperative alpha‐blockade or adrenergic preparation was administered. On the day of surgery, after anesthetic induction and endotracheal intubation, the patient experienced an abrupt rise in blood pressure to 168/118 mmHg and a pulse of 86/min. Invasive arterial monitoring was established, and the hypertensive crisis was managed intraoperatively with intravenous nitroglycerin and phentolamine. These hemodynamic fluctuations raised intraoperative suspicion of a functional paraganglioma, and a plasma metanephrine profile was sent immediately.

**FIGURE 3:**
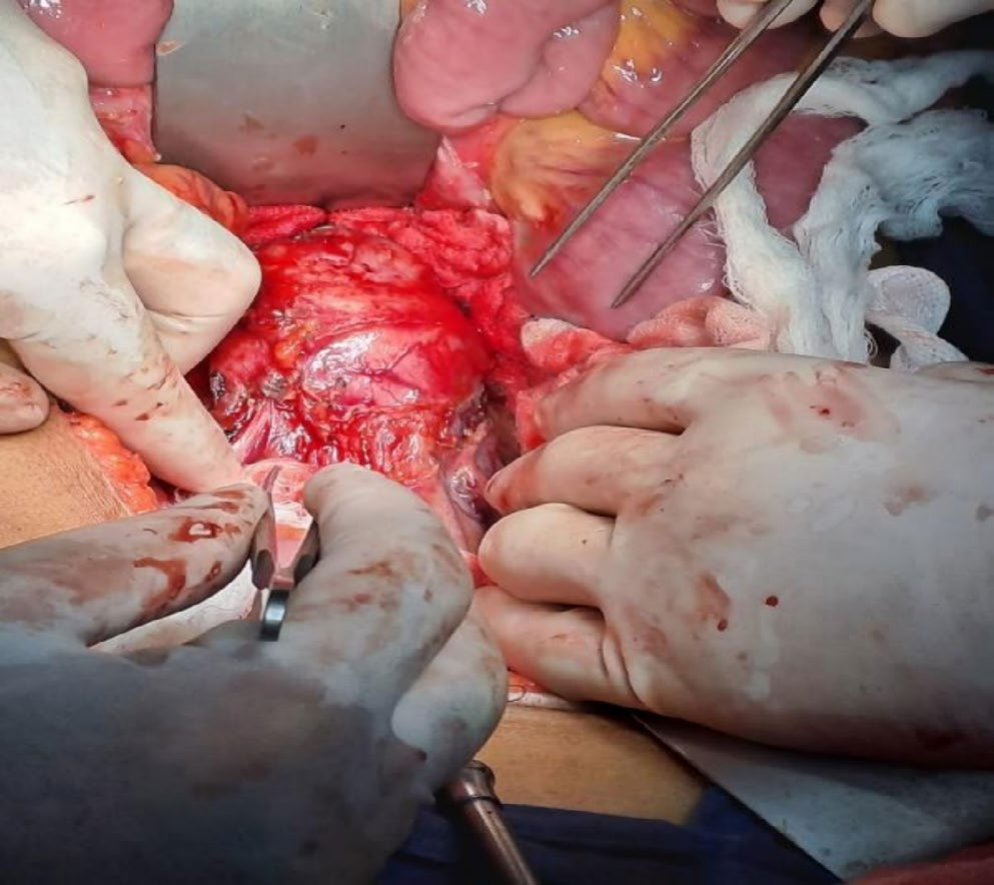
Exposure of mass during laparotomy.

**FIGURE 4:**
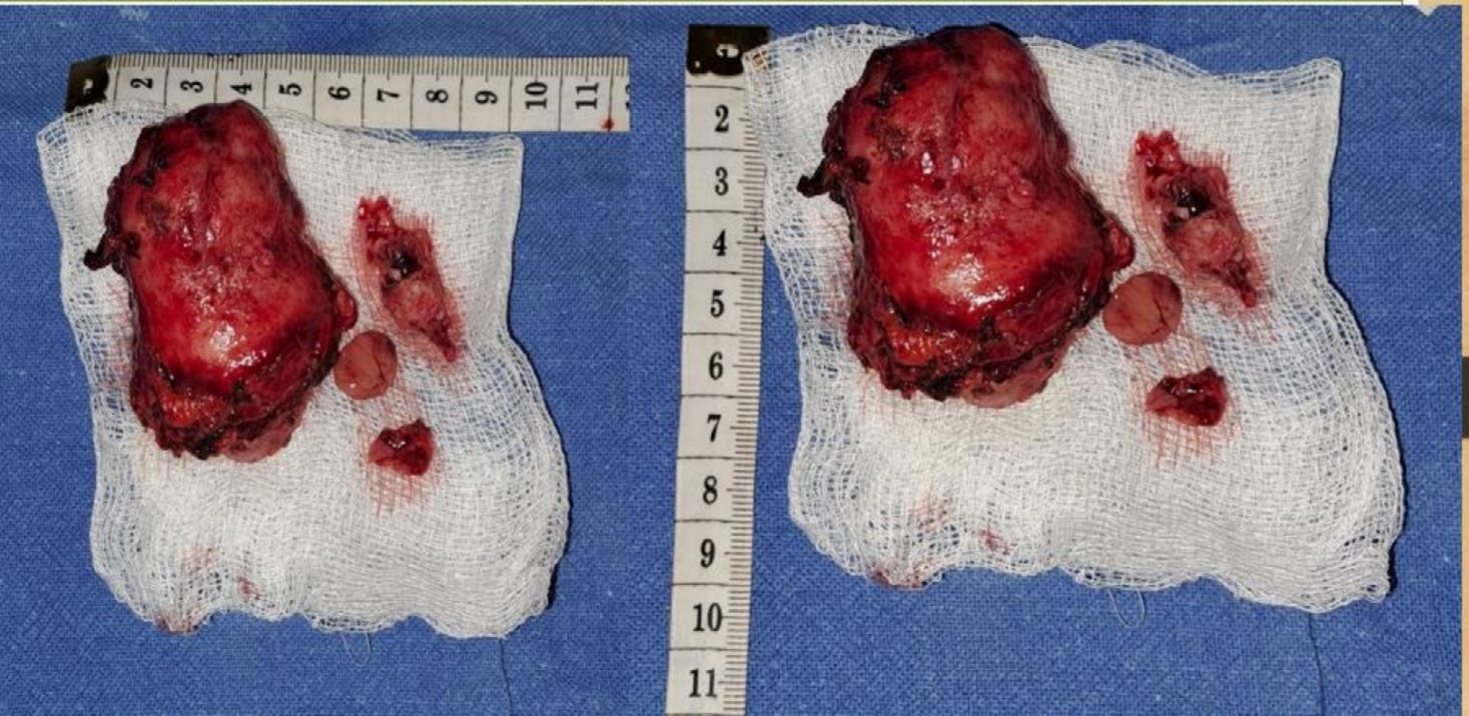
The excised tumor along with dimensions.

Exploratory laparotomy via midline incision revealed a firm, well‐encapsulated mass measuring approximately 6 × 4 cm situated at the aortic bifurcation as shown in Figure 3. The lesion was closely adherent to the anterior aortic wall, IVC, and adjacent duodenum. Meticulous dissection was carried out to mobilize the right colon and expose the retroperitoneum. The tumor was resected en bloc with ligation of feeding vessels and preservation of the surrounding vascular structures as shown in Figure 4. Throughout the procedure, intermittent hypertensive episodes occurred during tumor manipulation, which were effectively controlled with vasodilators and anesthetic depth modulation. Following tumor excision, the patient experienced a brief hypotensive period that was managed with norepinephrine infusion. A retroperitoneal drain was placed, and the patient was transferred to the intensive care unit for monitoring.

Postoperatively, the patient remained hemodynamically stable without further crises. Her drain output was minimal and removed on postoperative day two, and she resumed oral intake by day three. She was discharged on postoperative day four in satisfactory condition. The plasma metanephrine results, received postoperatively, revealed markedly elevated plasma‐free normetanephrine levels (1235.4 pg/mL; reference: 0–190 pg/mL) and borderline elevated metanephrine (94.4 pg/mL; reference: 0–90 pg/mL), confirming that the tumor had indeed been functional despite earlier normal urinary metanephrine levels.

Final histopathology of the excised tumor corroborated the diagnosis.

**FIGURE 5:**
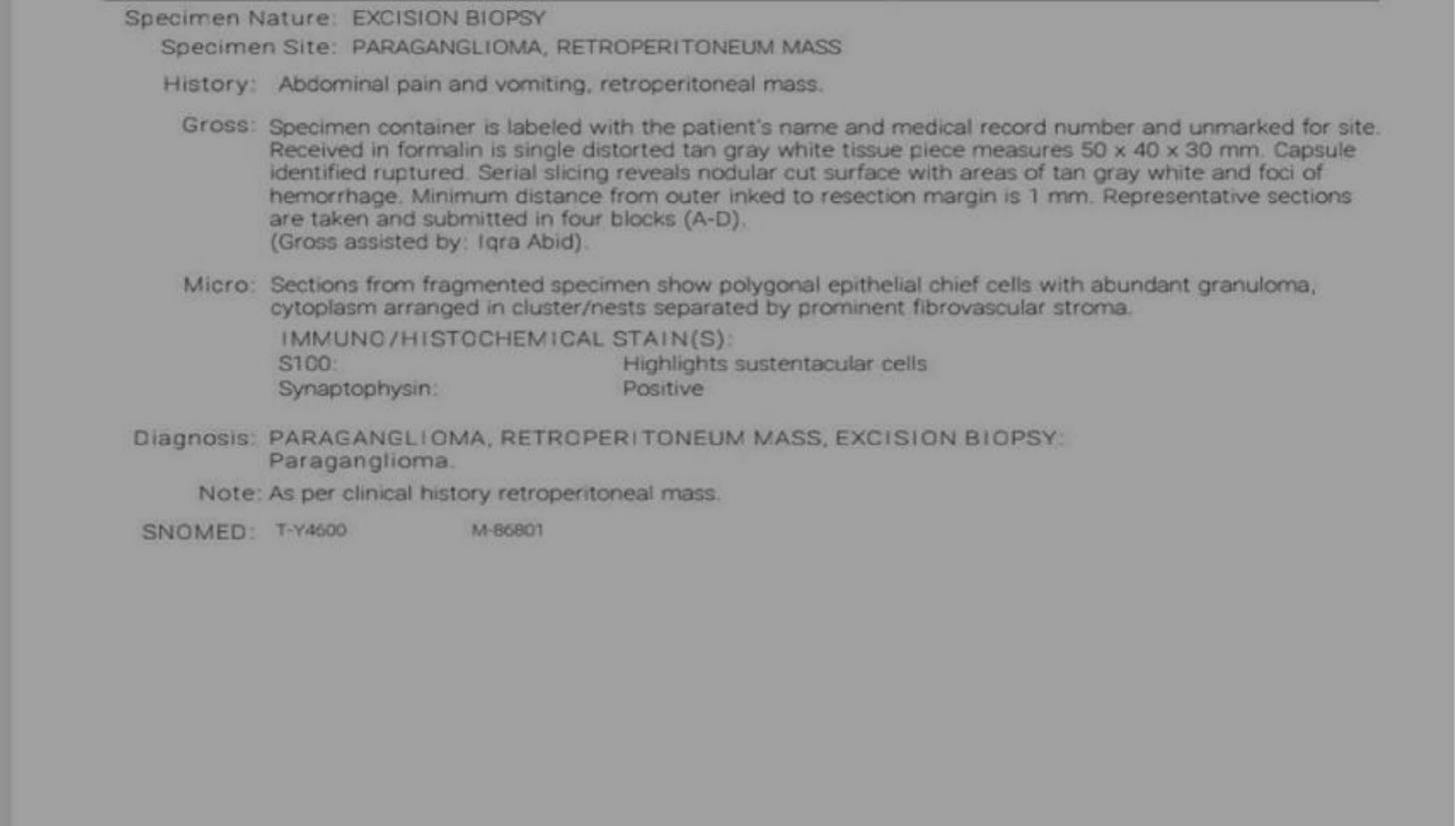
Histopathology report confirming the diagnosis.

The tumor showed well‐demarcated nests of polygonal chief cells with amphophilic cytoplasm and centrally located nuclei, with surrounding S‐100 positive sustentacular cells. Immunostaining was positive for chromogranin A, synaptophysin, and vimentin. There was no evidence of malignancy, necrosis, vascular invasion, or significant mitotic activity. The Ki‐67 proliferation index was < 1%, supporting a benign diagnosis as shown in Figure 5.

## 5 Outcome and Follow‐Up

The patient has remained well at 1‐ and 3–month follow‐ups, with complete resolution of abdominal symptoms, stable blood pressure, and no signs of recurrence on imaging. She is currently under surveillance with periodic catecholamine testing and radiologic monitoring.

## 6 Discussion

Extra‐adrenal paragangliomas are a rare and fascinating clinical entity. They originate from autonomic paraganglia—collections of neuroendocrine chromaffin cells distributed from the base of the skull to the pelvic region. In the abdomen, the organ of Zuckerkandl (a focal collection of chromaffin tissue at the aortic bifurcation) is known to be the most common site of extra‐adrenal paragangliomas [6]. Our patient's tumor was indeed located in this para‐aortic region, consistent with an Organ of Zuckerkandl paraganglioma. Paragangliomas may occur at any age but most commonly present in the third to fifth decades of life [3]. The occurrence in an adolescent (our patient was 19) is unusual and should prompt consideration of an underlying hereditary syndrome. Up to 30% of paragangliomas (and pheochromocytomas) are associated with germline mutations in susceptibility genes—most frequently SDH subunit mutations (SDHB, SDHD, etc.), as well as VHL, RET, NF1, among others [4]. Due to this high hereditary rate, current guidelines recommend genetic counseling and testing for all patients with pheochromocytomas/paragangliomas [4]. Our patient had no known family history of endocrine tumors; nonetheless, genetic testing would be prudent given her young age and tumor location to evaluate for conditions like SDH mutations or von Hippel–Lindau syndrome.

One of the central clinical considerations in paragangliomas is whether the tumor is functional or nonfunctional. Sympathetic paragangliomas (like those arising in the abdomen) are usually catecholamine‐producing; in fact, 90% or more will secrete excess norepinephrine and/or epinephrine, leading to symptoms or signs of adrenergic overactivity [3]. Typical manifestations include paroxysmal or sustained hypertension, headaches, palpitations, diaphoresis, pallor, and tremors—often precipitated by positional changes, anxiety, or tumor manipulation. On the other hand, about 10% of paragangliomas are considered clinically nonfunctional, meaning they do not produce significant catecholamine excess [3]. These silent tumors more often occur in locations like the head and neck (parasympathetic paragangliomas of the carotid body, vagal nerve, etc.), which characteristically do not secrete hormones. A nonfunctional abdominal paraganglioma (of sympathetic origin) is quite rare [3]. In our case, the patient's lack of catecholamine‐related symptoms and a normal screening test initially pointed to a nonfunctional tumor—a misleading impression that nearly had serious intraoperative consequences. This case underscores that even tumors deemed “silent” may have intermittent or provokable catecholamine release. It is known that some paragangliomas can be biochemically silent due to sporadic secretion or due to genetic factors; for example, certain SDHB‐mutated tumors have impaired catecholamine synthesis and may not elevate baseline plasma metanephrines [7]. Our patient's tumor likely secreted norepinephrine at low levels or intermittently, which was not captured by a single 24‐h urine test. However, the stress of induction anesthesia and surgical manipulation triggered a surge of catecholamine release, unmasking the tumor's functionality. Clinicians should therefore maintain a high index of suspicion: normal biochemical results do not completely rule out a functional paraganglioma if imaging and clinical context are suggestive. In such scenarios, some experts advocate proceeding with preoperative adrenergic blockade as a precaution, because the risk of a missed functional tumor (and an unprepared hypertensive crisis) can be catastrophic [4, 6].

Proper biochemical evaluation is a cornerstone of diagnosing pheochromocytomas and paragangliomas. The recommended tests are those that detect excess catecholamine production. Plasma‐free metanephrines (metanephrine and normetanephrine) have the highest sensitivity of any single test, reported at approximately 97%–99% in various studies [5]. This high sensitivity makes plasma‐free metanephrines the preferred initial screening test for PPGLs in many guidelines. In our patient's case, unfortunately, this test was done only retrospectively (and indeed came back positive). Instead, a 24‐h urinary total metanephrine was used initially; while urinary fractionated metanephrines are also a valid diagnostic test (with decent specificity), they can yield false‐negative results in about 1%–2% of cases [5]. If clinical suspicion remains high despite a negative test, it is advisable to either repeat the test, obtain plasma fractionated metanephrines, or proceed empirically as if the tumor is functional. Our patient's scenario illustrates the limitation of a single normal test—especially since her tumor predominantly produced norepinephrine (reflected by the isolated huge rise in normetanephrine), which might have been missed if the urine assay measured primarily metanephrine (an epinephrine metabolite). In summary, comprehensive biochemical testing (including both metanephrine and normetanephrine levels, preferably plasma‐free levels) is critical when evaluating a suspected paraganglioma. Moreover, one must consider that medications (e.g., beta‐blockers, antidepressants), diet, and tumor secretory patterns can affect test results and interpret findings in the full clinical context.

Imaging is the next key component in the workup, aimed at localizing the tumor and assessing its extent. In our patient, both CT and MRI played complementary roles. CT scan is often the first‐choice imaging modality for abdominal paragangliomas due to its wide availability and excellent spatial resolution [6]. Paragangliomas on CT typically appear as well‐defined retroperitoneal masses that intensely enhance with contrast, reflecting their hypervascular nature, and they may exhibit areas of central necrosis or hemorrhage if large [8]. Our patient's tumor demonstrated exactly this pattern on CT (heterogeneous enhancement with a necrotic core and a prominent vascular supply). MRI can provide additional characterization; paragangliomas classically show very high signal intensity on T2‐weighted images (the so‐called “light‐bulb bright” lesion) and bright enhancement on T1 post‐contrast images [9]. The MRI in this case confirmed the lesion's hyperintense T2 signal and relationship to major vessels. While CT/MRI are usually sufficient for preoperative planning, functional imaging can be valuable in certain scenarios—for example, detecting multifocal disease or metastases. ^123^I‐metaiodobenzylguanidine (MIBG) scintigraphy is a nuclear medicine scan that specifically concentrates in adrenergic tissue and can confirm that an adrenal/extra‐adrenal mass is catecholamine‐producing. Newer techniques like [^68^Ga]‐DOTATATE PET/CT (which targets somatostatin receptors) and ^18^F‐FDG PET are highly sensitive in mapping paraganglioma lesions throughout the body [6]. In a patient with known paraganglioma, these functional imaging modalities are often used to rule out multiple tumors or metastatic spread. In our resource‐limited setting, such scans were not obtained prior to surgery; fortunately, post‐op evaluation did not find evidence of additional tumors. Recent advances in the therapeutic management of paragangliomas have expanded beyond surgical resection, particularly for unresectable, recurrent, or metastatic disease. Among these, peptide receptor radionuclide therapy (PRRT) has emerged as a promising targeted treatment modality. PRRT utilizes radiolabeled somatostatin analogs (such as ^177^Lu‐DOTATATE) that bind to somatostatin receptors, which are highly expressed in many neuroendocrine tumors, including paragangliomas. This allows for selective delivery of radiation to tumor cells while sparing surrounding tissues [10]. Clinical studies have demonstrated that PRRT can achieve disease stabilization, symptomatic improvement, and, in some cases, partial tumor regression with an acceptable safety profile. Moreover, PRRT is particularly valuable in patients with somatostatin receptor–positive tumors identified on functional imaging (e.g., Ga‐68 DOTATATE PET/CT). In addition to PRRT, other systemic options such as high‐specific‐activity MIBG therapy, tyrosine kinase inhibitors, and chemotherapy may be considered depending on tumor behavior and genetic background. These evolving therapeutic strategies highlight the importance of a personalized, multidisciplinary approach in managing paragangliomas, especially in advanced disease settings [11].

Surgical resection is the definitive treatment for localized paragangliomas. Complete removal of the tumor offers the best chance of cure or long‐term control, whether the tumor is functional or not. However, surgery carries unique challenges, especially for tumors in tricky locations like the aortocaval region. Vascular proximity in our case necessitated meticulous dissection to avoid major hemorrhage or vascular injury. An important consideration is the surgical approach: many adrenal pheochromocytomas and some extra‐adrenal paragangliomas can be removed laparoscopically, which offers a faster recovery. There are reports of successful laparoscopic resection of retroperitoneal paragangliomas even up to ~6 cm in size [3]. In our patient's case, given the tumor's adherence to great vessels and the anticipated hemodynamic volatility, an open approach was deemed safer for maximal control. Intraoperatively, anesthetic management is crucial. Blood pressure lability is common during induction, intubation, and especially during tumor handling, due to catecholamine surges. Our patient's intraoperative hypertension was managed with titrated vasodilators; in cases known to be functional, an infusion of a short‐acting alpha blocker (e.g., phentolamine) or nitroprusside is often used to maintain stability. Conversely, after the tumor is removed, patients can have rebound hypotension as circulating catecholamines plummet—sometimes necessitating brief vasopressor support. Close communication between the surgical and anesthesia teams is essential to navigate these shifts. Fortunately, our center's intraoperative monitoring and management allowed the surgery to proceed safely despite the unexpected hypertension. The postoperative course for this patient was uncomplicated, which is consistent with adequate tumor resection and the absence of residual catecholamine effect.

On pathology, our case demonstrated features typical of paraganglioma, which helped confirm the diagnosis. Paragangliomas are composed of chief cells arranged in nests (zellballen) surrounded by sustentacular cells. The chief cells have characteristic granular amphophilic cytoplasm and stippled (“salt‐and‐pepper”) chromatin, due to their neuroendocrine secretory granules [6]. Immunohistochemical staining is usually positive for general neuroendocrine markers—chromogranin A and synaptophysin are expressed in essentially all paragangliomas [6]. The sustentacular cells (which are modified Schwann cells) typically express S‐100 protein, as seen in our patient's tumor. These features distinguish paragangliomas from other potential mimics like adrenal cortical tumors or extra‐adrenal neurogenic tumors. Notably, functional and nonfunctional paragangliomas are histologically indistinguishable; the difference is only in clinical and biochemical behavior. There are no reliable histopathological criteria to determine malignancy in paragangliomas. Unlike many other tumors, cytologic atypia or mitotic activity does not necessarily correlate with malignant potential. The definition of malignant paraganglioma is the presence of metastases (tumor deposits in sites where paraganglial tissue is normally absent, such as bone, liver, lung, lymph nodes) [12]. In our patient's tumor, no metastases were evident at presentation, and the surgical pathology showed no local invasion, suggesting a benign behavior. Nonetheless, long‐term follow‐up is warranted.

### 6.1 Prognosis and Follow‐Up

Extra‐adrenal paragangliomas are potentially curable with surgery, but patients require life‐long follow‐up because of risks of recurrence or malignancy. Published series indicate that even after complete resection, tumor recurrence can occur in about 3%–16% of patients over time [3]. Recurrence may present as local regrowth or as new lesions in other paraganglia (especially in hereditary cases). Our patient will need periodic re‐evaluation, including plasma or urine metanephrine testing and imaging, to detect any recurrence early. Malignant transformation (metastatic disease) is seen in a subset of cases—historically around 10% of pheochromocytomas, but extra‐adrenal paragangliomas may have a higher rate of malignant behavior (some reports suggest 20% or more) [9]. Certain factors, such as large tumor size, extra‐adrenal location, and SDHB mutation status, are associated with an increased risk of malignancy [4, 9]. In the absence of definitive histologic markers, close surveillance is the strategy for managing this risk. If malignancy does occur, treatment may involve surgery for localized metastases, systemic therapies, or targeted radionuclide therapy (e.g., MIBG therapy or peptide receptor radionuclide therapy). On a positive note, our patient's outcome thus far is excellent—her symptoms resolved after tumor removal, and she has returned to normal life. This case emphasizes the importance of a multidisciplinary approach (involving surgeons, endocrinologists, anesthesiologists, geneticists, and others) in the management of paragangliomas to ensure safe perioperative care and appropriate follow‐up.

## 7 Conclusion

This case highlights the diagnostic challenges posed by apparently nonfunctional paragangliomas, especially when biochemical tests are falsely negative. Clinicians should maintain a high index of suspicion and consider perioperative preparation even in the absence of classical symptoms or positive initial labs, as undiagnosed functional tumors can cause significant intraoperative morbidity.

## Author Contributions

**Abdul Basit:** conceptualization, validation, writing – original draft, writing – review and editing. **Tooba Yaqoob:** visualization, writing – original draft, writing – review and editing. **Muhammad Usman Ali Khan:** writing – original draft, writing – review and editing. **Eyman Farrukh:** writing – original draft, writing – review and editing. **Abdullah Iftikhar:** writing – original draft, writing – review and editing. **Majeed Haq:** resources, supervision.

## Funding

The authors have nothing to report.

## Ethics Statement

The authors have nothing to report.

## Consent

A written informed consent was obtained from the patient to “publish” this report in accordance with the journal's patient consent policy.

## Conflicts of Interest

The authors declare no conflicts of interest.

## Data Availability

Data is available on request.
